# Overexpression of CD39 in hepatocellular carcinoma is an independent indicator of poor outcome after radical resection

**DOI:** 10.1097/MD.0000000000004989

**Published:** 2016-10-07

**Authors:** Xiao-Yan Cai, Xiao-Chun Ni, Yong Yi, Hong-Wei He, Jia-Xing Wang, Yi-Peng Fu, Jian Sun, Jian Zhou, Yun-Feng Cheng, Jian-Jun Jin, Jia Fan, Shuang-Jian Qiu

**Affiliations:** a Liver Cancer Institute, Zhongshan Hospital, Fudan University, Key Laboratory for Carcinogenesis & Cancer Invasion, The Chinese Ministry of Education; b Department of General Surgery, Gongli Hospital; c Biomedical Research Center, Zhongshan Hospital, Fudan University, Shanghai, People's Republic of China.

**Keywords:** CD39, hepatocellular carcinoma, prognosis

## Abstract

Nucleoside triphosphate diphosphohydrolase-1 (ENTPD1/CD39) is the rate-limiting enzyme in a cascade leading to the generation of immunosuppressive adenosine and plays an important role in tumor progression. This study aimed to evaluate the expression of CD39 and CD39^+^Foxp3^+^ regulatory T cells (Tregs) and to determine their prognostic role in patients with hepatocellular carcinoma (HCC) after radical resection.

Immunohistochemistry (IHC) and double IHC were used to analyze CD39 expression or the expression of CD39 and Foxp3 in a cohort of 324 HCC patients who underwent curative resection. The quantification of CD39 expression levels was determined using a computerized image analysis system and was evaluated by mean optical density (MOD), which corresponded to the positive staining intensity of CD39. The number of positive Foxp3 cells and both CD39 and Foxp3 positive cells in each 1-mm-diameter cylinder were counted under high-power magnification (×400). The “minimum *P* value” approach was used to obtain the optimal cutoff value for the best separation between groups of patients in relation to time to recurrence (TTR) or overall survival (OS). The expression of CD39 in HCC cell lines with stepwise metastatic potential and in human umbilical vein endothelial cells was determined by reverse transcription-polymerase chain reaction, Western blotting, and immunofluorescence. The SPSS 17.0 statistical package was used for statistics.

CD39 was principally expressed on vascular endothelial cells, macrophagocytes, Tregs, and tumor cells in HCC. Compared with paired peritumoral tissues, tumoral tissues had a significantly higher expression level of CD39 (*P* < 0.0001). Overexpression of tumoral CD39 was related to increased tumor recurrence and shortened overall survival. Furthermore, the expression level of peritumoral CD39 showed a prognostic role in TTR and OS. Double IHC showed that tumoral tissues had significantly higher Foxp3^+^Tregs and CD39^+^Foxp3^+^Tregs count per 1 mm core (14.1659 vs 4.9877, *P* = 0.001; 11.5254 vs 3.3930, *P* < 0.001) and a higher CD39^+^Foxp3^+^/Foxp3^+^ ratio compared with paired peritumoral tissues. CD39^+^Foxp3^+^Tregs were a better prognosticator than CD39^+^Tregs for TTR.

Overexpression of CD39 protein in HCC was an independent predictor of poor outcome after radical resection. The CD39^+^Foxp3^+^Tregs count added prognostic power to Foxp3^+^Tregs, providing a potential target for tumor immunotherapy.

## Abstract 2

Supplemental Digital Content is available in the text

## 1 Introduction

Recent years have witnessed the renaissance of the tumor immunosurveillance concept and expansion of the initial notion of “immunoediting,” of which the tumor escape phase attracts the most interest in researchers. The escape phase is the final phase of the process, in which tumor cells can grow quickly and become clinically apparent, establishing an immunosuppressive tumor microenvironment.^[1]^ The mechanisms of tumor escape may include: reduced immune recognition, increased resistance or survival, or development of an immunosuppressive tumor microenvironment. From an immunobiologic perspective, tumor local immune response comprises 2 arms: antitumor immunity such as CD8^+^ T cells, natural killer (NK) cells, and protumor factors such as regulatory T cells (Tregs) and tumor-derived repressive factors.^[2]^ The balance between antitumor and protumor factors is important for tumor recurrence. In the past few years, our institute has witnessed a growing list of moieties that contribute to tumor-induced immunosuppression, such as CD151, PD-L1, CXCR6, HLA-G, hypoxia-inducible factor-1 alpha, B7-H3, galectin-1, and macrophage colony-stimulating factors.^[3–10]^ Recently, a new immunoregulatory molecule, nucleoside triphosphate diphosphohydrolase-1 (ENTPD1, CD39), which is the dominant ectonucleotidase expressed on numerous different types of cells such as normal leukocytes,^[11]^ endothelial cells,^[12,13]^ and Tregs,^[14,15]^ regulating extracellular nucleotide/nucleoside concentrations by scavenging nucleotides to ultimately generate adenosine, was identified. It has also been described as a new functional surface marker for Tregs, which are regarded as a poor predictor for the outcome of hepatocellular carcinoma (HCC).^[6,16]^ This protein can catalyze the sequential hydrolysis of extracellular adenosine triphosphate (ATP), known to boost immune responses and may also contribute directly to cancer cell death in the tumor microenvironment, to adenosine monophosphate (AMP), which is then further degraded to anti-inflammatory adenosine by CD73/ecto-5′-nucleotidase.

Overexpression of CD39 has been observed in many human cancer types such as melanoma,^[17]^ leukemia,^[18]^ pancreatic cancer,^[19]^ colon cancer,^[20]^ and ovarian cancer.^[21]^ Lower levels of CD39 mRNA in colorectal cancer appear to be associated with longer survival and could be linked to less invasive tumors.^[20]^ Nevertheless, to date, there has been no comprehensive description of the levels of CD39 expression in tissue samples of human HCC collected from a representative and appropriately large cohort of patients, and its prognostic role has not been described.

There is growing evidence to indicate that HCC is typically associated with chronic inflammatory states, which are linked to immune dysregulation, disordered metabolism, and aberrant cell proliferation. In human and murine liver specimens, CD39 has been observed to be strongly expressed in Kupffer cells and endothelial cells (ECs) of muscularized vessels in the liver.^[22]^ CD39 expression by ECs may directly protect tumor cells from high levels of extracellular ATP, which directly limits tumor cell growth and these antitumor effects could be mitigated by the provision of CD39 or by the intrinsic EC expression of CD39.^[23]^ CD39 expression in Tregs inhibits natural killer (NK) cell activity and is necessary for the growth of metastatic tumors in the liver.^[24]^

In the present study, we evaluated the expression of CD39 and Foxp3 in a large cohort of 324 HCC patients using double immunohistochemistry (IHC). We found high expression of CD39 in liver tumoral tissue was related to poor prognosis in HCC patients after resection. The Foxp3^+^ and CD39^+^Foxp3^+^ cell counts in tumoral tissue were higher than those in peritumoral tissue, and were related to time to recurrence (TTR) and overall survival (OS).

## 2 Materials and methods

### 2.1 Patients and tissue microarray

Total of 324 patients with HCC who underwent curative resection, defined as complete macroscopic removal of the tumor, between 2007 and 2008 at the Liver Cancer Institute of Fudan University (Shanghai, China) Zhongshan Hospital (Shanghai, China) were enrolled. Tissue microarray (TMA) involved 324 patients with informed consent and approval was obtained. For each case, three 1-mm cores from 2 different areas, the tumor center and non-tumor tissues (over 1 cm away from the tumor margin), were obtained to ensure reproducibility of staining and placed on 3-aminopropyltriethoxysilane-coated slides. The inclusion criteria were as follows: confirmed pathologic diagnosis of HCC; no preoperative anticancer treatment or signs of distant metastasis; integrated clinicopathological characteristics and postoperative follow-up data, which were described previously.^[16]^ After surgery, patients with a high risk of recurrence, such as vascularinvasion and spreading nodules, were treated with prophylactic transcatheterarterial chemoembolization (doxorubicin, cisplatin, fluorouracil, and iodized oil; 1–3 courses). TTR and OS were defined as the interval between surgery and recurrence and between surgery and death or the last observation for surviving patients, which were censored at last follow-up (December 31st, 2013).

### 2.2 Tissue immunohistochemical staining and evaluation

Immunohistochemical double staining and the MultiVision Polymer Detection System (Thermo Scientific, Rochester, NY) were used with the primary antibody cocktail of rabbit anti-human CD39 (1:200; Sigma-Aldrich, St Louis, MO) and mouse anti-human Foxp3 (1:200; BioLegend, San Diego, CA). The details were seen in Supplementary File. Positive anti-human rabbit or mouse primary antibody staining was blue or red, respectively. When both showed a positive result, the staining was purple and was visualized using a computerized image system composed of a camera connected to an OLYMPUS U-CAMD3 microscope. The quantification of CD39 expression levels was determined using a computerized image analysis system. Images using low-power magnification (×100) fields were captured in each 1-mm-diameter cylinder. Positive staining was evaluated by mean optical density (MOD), which corresponded to the positive staining intensity of CD39. Under high-power magnification (×400), the number of positive Foxp3 cells and both CD39 and Foxp3 positive cells in each 1-mm-diameter cylinder were counted by 2 experienced pathologists who were blinded to the clinicopathologic data of the patients and calculated as the mean count of the triplicate values (cells/spot). The other 2 primary antibody cocktails containing rabbit anti-human CD31 (1:50, R&D, Minneapolis, MN) plus mouse anti-human CD39 and rabbit anti-human CD39 plus mouse anti-human CD68 (1:1000, Abcam, Cambridge, MA) were tautologically applied as double staining in selected samples, as described above.

### 2.3 Cell lines

Five human HCC cell lines and a normal hepatocyte line were used, which included MHCC97H, MHCC97L, SMCC-7721, Huh-7, HepG2, and Changliver, respectively. The first 2 HCC cell lines with stepwise pulmonary metastatic potential (MHCC97H and MHCC97L) were established at our institute.

### 2.4 Western blot analysis

Immunoblotting was carried out as previously described.^[25]^ In brief, approximately 30 μg of protein extracted from 6 cell lines was separated by SDS-PAGE, the protein was then transferred to a polyvinylidene fluoride membrane (Millipore), and membrane-bound CD39 was detected using rabbit anti-human CD39 (1:1000, Sigma-Aldrich). GAPDH (1:5000, Kangcheng, Shanghai) was used as an internal control.

### 2.5 Immunofluorescence assay

CD39 expression in human umbilical vein endothelial cells was detected by immunofluorescence assay. Cells cultured on glass slides were fixed by acetone for 15 minutes. After treating with 0.2% Triton X-100 for 2 minutes, the fixed cells were blocked with bovine serum albumin and stained with rabbit anti-human CD39 monoclonal antibody (1:200) at 4°C overnight and DyLightTM 488-Conjugated Goat Anti-Rabbit IgG at 37°C for 30 minutes. A negative control (primary antibody omitted) was included on each slide. After rinsing in PBS, the slides were counterstained with 4,6-diamidino-2-phenylindole (Vector Laboratories, Inc, Burlingame, CA) and examined under a fluorescent microscope (Olympus BX 40).

### 2.6 Statistical analysis

The SPSS 17.0 statistical package was used. The *χ*^2^ test and paired *t* test were carried out as appropriate. Univariate analyses were performed using the Kaplan–Meier method and compared using the log-rank test. Cox multivariate analysis was used to adjust for potentially confounding variables and to determine the independent prognostic factors. The “minimum *P* value” approach was used to obtain the optimal cutoff value for the best separation between groups of patients in relation to TTR or OS. Significance was accepted when *P* < 0.05.

## 3 Results

### 3.1 Characteristics of the patient cohort

The clinicopathological characteristics of the patients were shown in Supplementary Table 1. The median follow-up period was 61.03 months (range 2–82.33 months; SD 26.09 months). At the last follow-up (December 31st, 2013), 196 patients had HCC recurrence, and 142 patients died of recurrence. The 1-, 3-, and 5-year cumulative recurrence and survival rates (in brackets) were 35% (83%), 55% (66%), and 61% (55%), respectively.

### 3.2 Expression of CD39 in HCC

**Figure 1:**
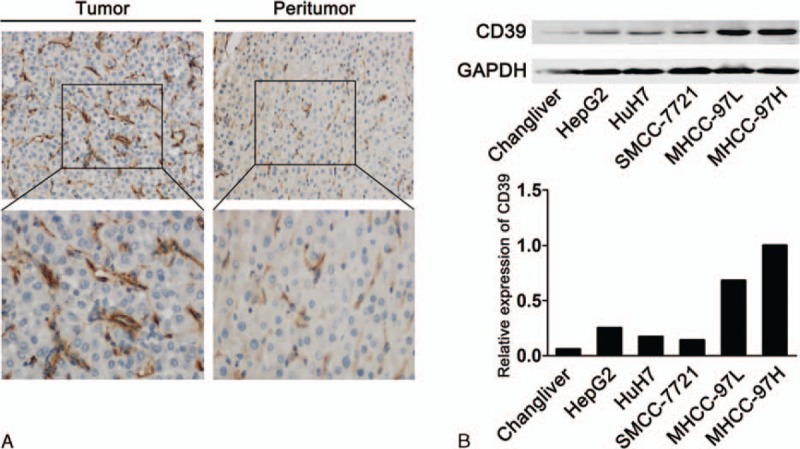
Expression of CD39 in hepatocellular carcinoma (HCC) tissues and cell lines. (A) Representative immunohistochemical staining of CD39 in tumoral and paired peritumoral tissues. The expression of CD39 in tumor was higher than that in paired peritumoral tissues (A and B, magnification 200× and 400×). (B) The expression of CD39 in 5 HCC cell lines with stepwise metastatic potential and in normal hepatocytes was determined by immunoblotting.

Positive CD39 staining was seen as brown, and was principally scattered in the tumoral or peritumoral mesenchyma and parenchyma, of which tumor cells and vascular endothelial cells were obviously positive (Fig. 1A). To confirm this finding, 5 human HCC cell lines and a normal hepatocyte line were used to determine the expression of CD39. All the cell lines expressed CD39; the highest and lowest expression was found in MHCC97H and Changliver cells, respectively (Fig. 1B). Furthermore, the expression level of CD39 was closely associated with the pulmonary metastatic potential.

**Figure 2:**
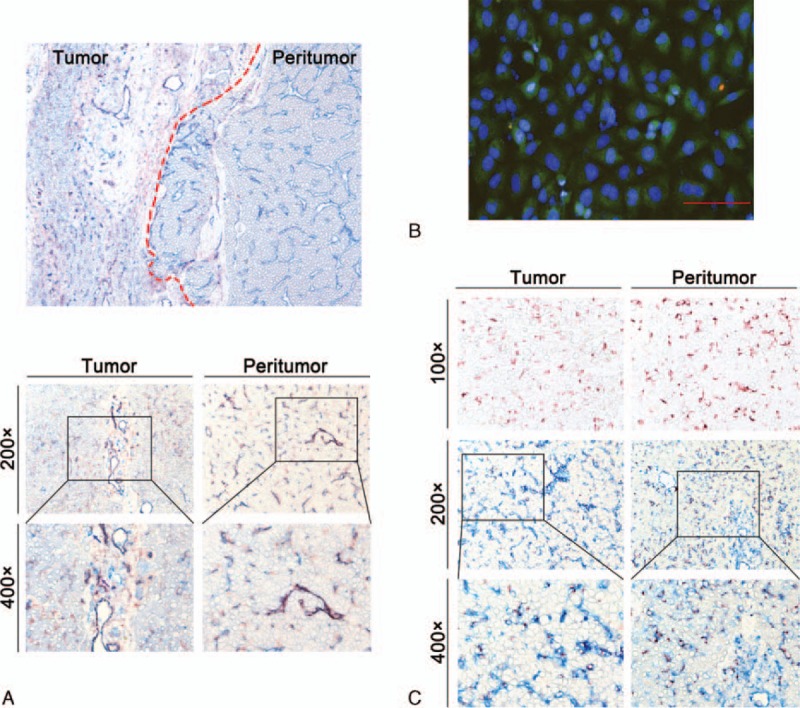
The expression of CD39 on various cells. (A) Double immunohistochemical staining of CD31 (blue) and CD39 (red) in tumor and paired peritumoral tissues. There was a large proportion of double positive cells (purple) in tumor and paired peritumoral tissues (magnification 100×, 200×, 400×). (B) CD39 expression in human umbilical vein endothelial cells by immunofluorescence assay (scale bar = 50 μm). (C) Immunohistochemical staining of CD68 (red) in tumor and paired peritumoral tissues (magnification 100×, 200×, and 400×). Double immunohistochemical staining of CD39 (blue), CD68 (red), and double positive cells (purple) in tumor and paired peritumoral tissues (magnification 200× and 400×).

We used double staining with the primary antibody cocktail of CD31 plus CD39 and CD39 plus CD68 and found CD39^+^ cells (red) were more in tumor than in peritumoral tissue (Fig. 2A, ×100). In both tumor and peritumoral tissue, the double positive areas were clearly shown in purple, indicating that most CD39 positive cells were located in vascular endothelial cells, especially in tumor (Fig. 2A). The immunofluorescence assay demonstrated that CD39 was also expressed in human umbilical vein endothelial cells (Fig. 2B). Some of the CD68 positive cells were purple, which indicated that the macrophagocytes in HCC expressed CD39 (Fig. 2C).

### 3.3 Correlations between CD39 expression and clinicopathological characteristics of HCC

By using the “minimum *P* value” approach, the MOD values of 0.04594 and 0.02149 were the best cutoff values for intratumoral and peritumoral CD39 expression, respectively. The clinicopathological characteristics of HCC were analyzed in relation to the low or high level of intratumoral and peritumoral CD39 expression. As shown in Table 1, the expression of CD39 in peritumoral tissue was positively correlated with large tumor, tumor vascular invasion, and advanced BCLC stages.

**Table 1:**
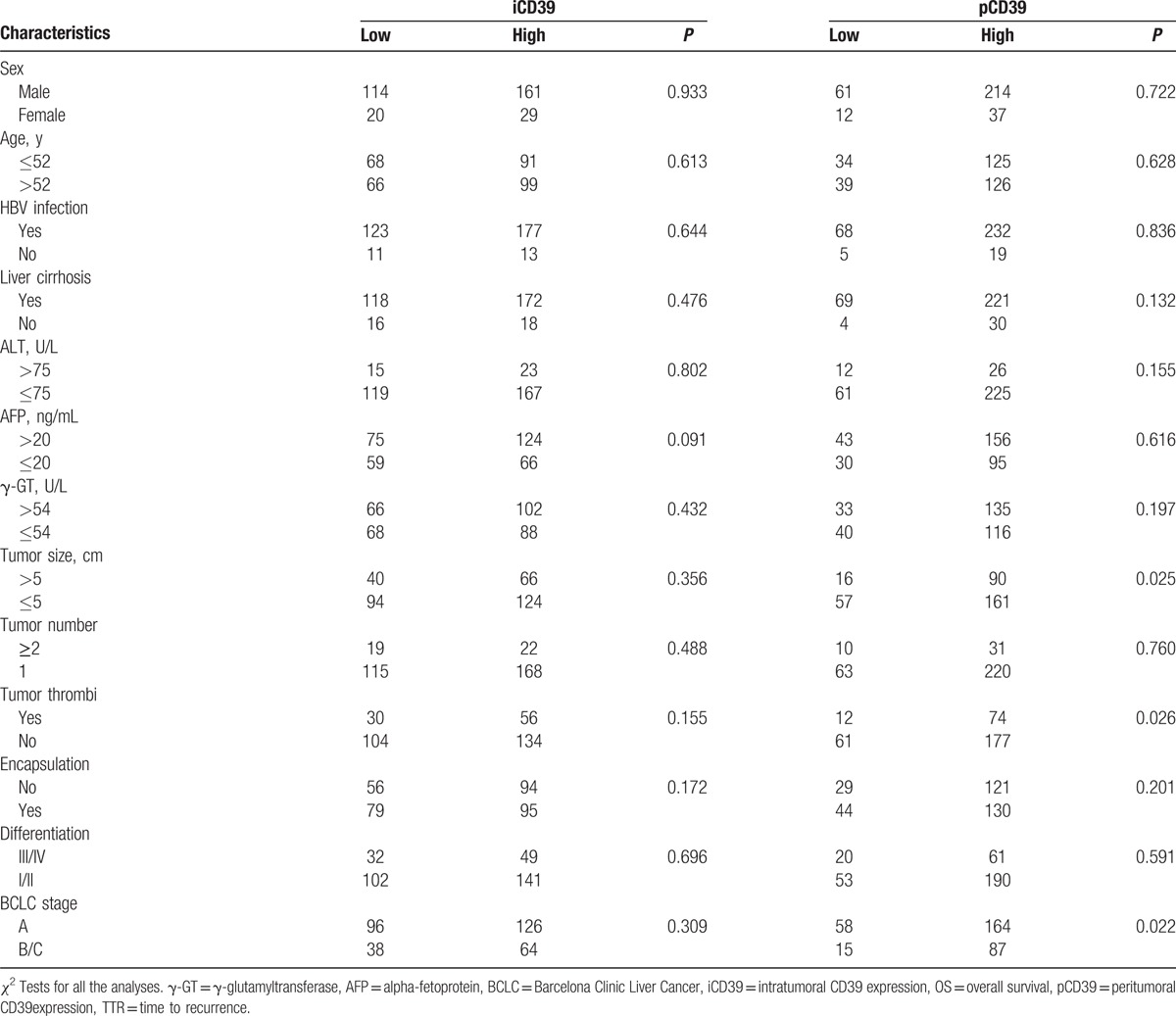
Correlations between clinicopathologic characteristics and CD39 expression.

### 3.4 Prognostic significance of CD39 expression in HCC

**Figure 3:**
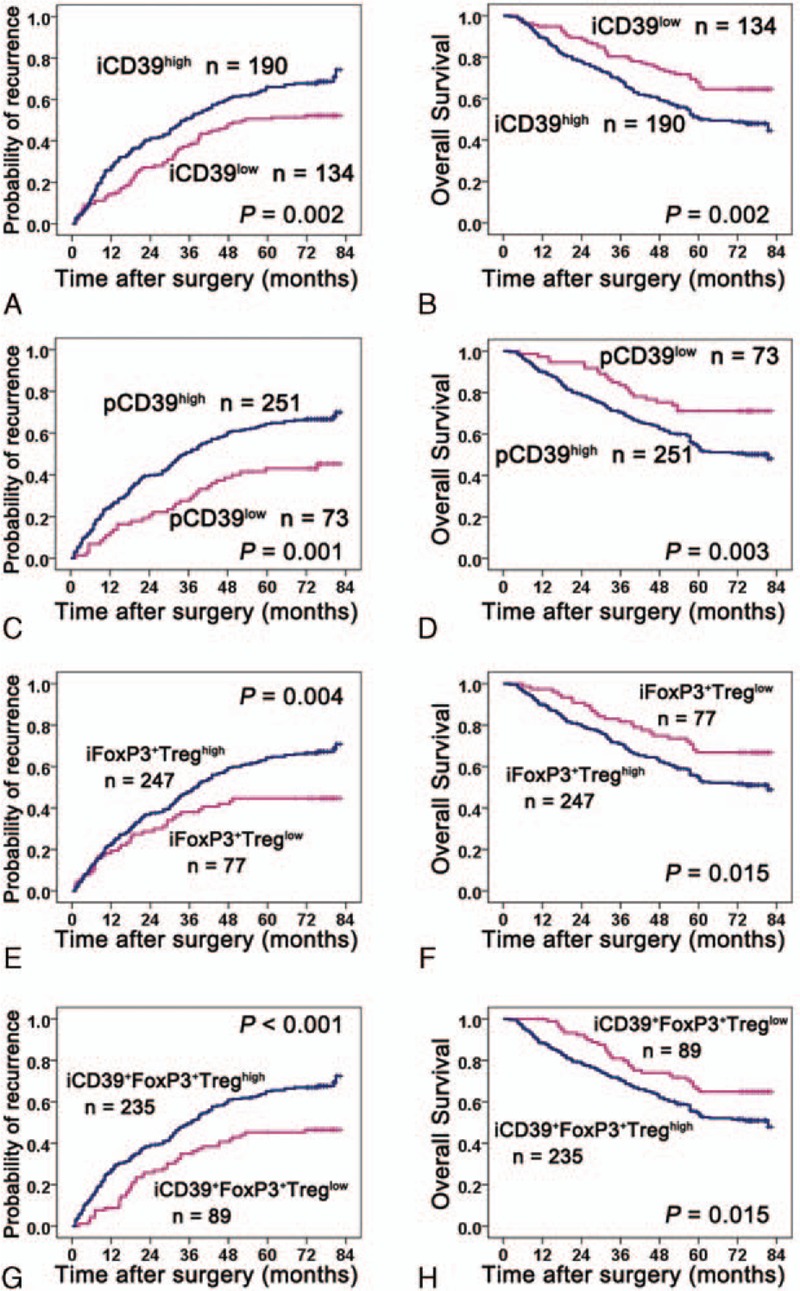
Kaplan–Meier analysis of TTR and OS in relation to expression levels of CD39 and the Foxp3^+^ and CD39^+^Foxp3^+^Tregs count. Univariate analyses of the relationship between **t**he expression level of CD39 and TTR or OS in tumoral (A and B) and peritumor tissues (C and D), respectively. (E and F) Univariate analyses of the relationship between the Foxp3^+^Tregs count in tumor and TTR or OS. (G and H) Univariate analyses of the relationship between the CD39^+^Foxp3^+^Tregs count in tumor and TTR or OS. iCD39 = intratumoral CD39, iCD39^+^FoxP3^+^Treg = intratumoral CD39^+^FoxP3^+^Treg, iFoxP3^+^Treg = intratumoral FoxP3^+^Treg, OS = overall survival, pCD39 = peritumoral CD39, TTR = time to recurrence.

On univariate analysis, the levels of CD39 expression were related to both TTR and OS in tumoral and peritumoral tissue (Table 2, Fig. 3A–D). We also performed multivariate Cox proportional hazard regression analyses to determine the relationship between the level of CD39 expression and TTR or OS and showed that the level of tumoral or peritumoral CD39 expression was independently related to both TTR and OS.

**Table 2:**
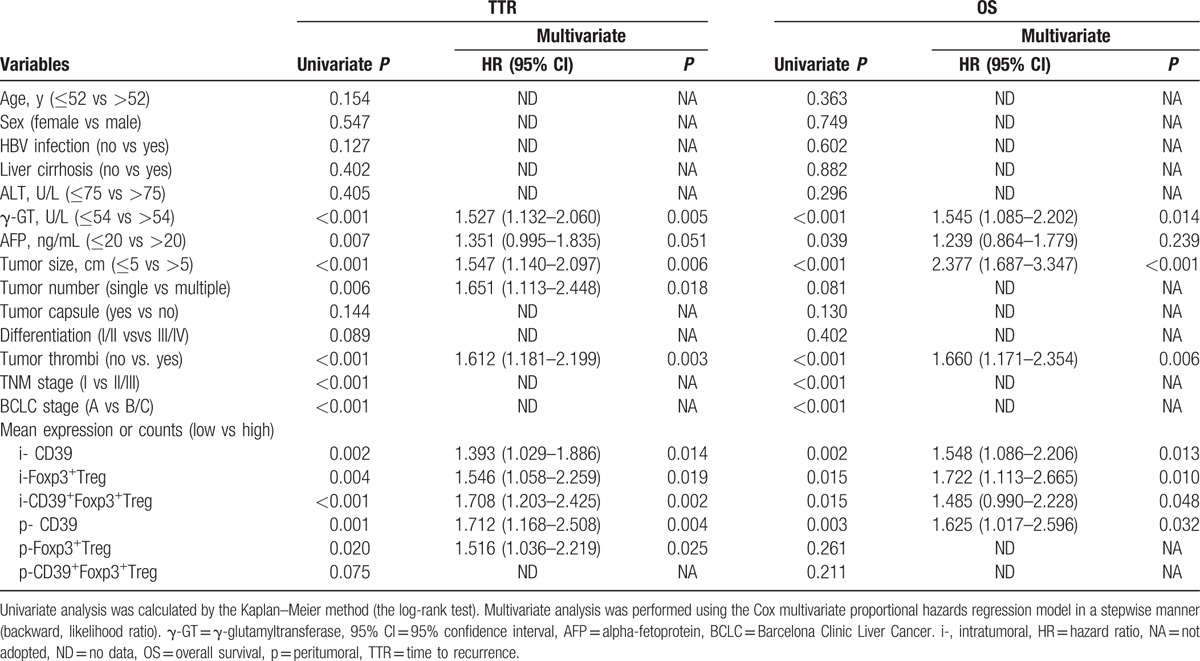
Univariate and multivariate analyses of CD39^+^ and CD39^+^Foxp3^+^Tregs associated with recurrence and survival.

### 3.5 Immunohistochemical expression of Foxp3^+^ and CD39^+^Foxp3^+^Treg cells in HCC

**Figure 4:**
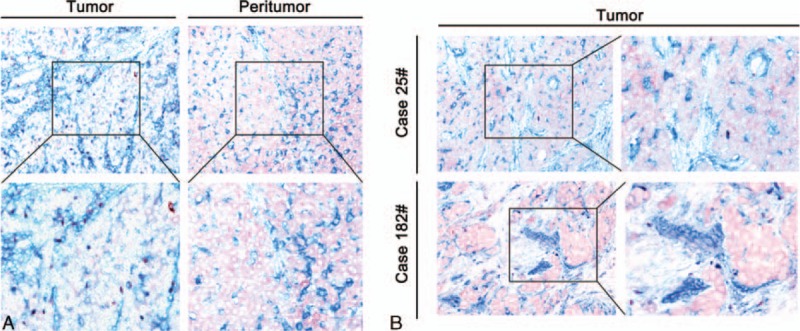
Representative immunohistochemical staining of CD39 and Foxp3. (A) Either FoxP3^+^ or CD39^+^FoxP3^+^Treg counts were higher than in the peritumoral counterparts. (B) Two representative cases of tumoral tissues are shown (magnification 200× and 400×). CD39^+^ cells are blue, Foxp3^+^ cells are red and double positive cells are purple.

Foxp3^**+**^ and CD39^**+**^Foxp3^**+**^Treg cells, which were seen as red and purple, respectively, were principally scattered in the mesenchyma and parenchyma (Fig. 4). Compared with paired peritumoral tissues, tumoral tissues had significantly higher Treg counts per 1-mm core (14.1659 vs 4.9877, *P* = 0.001; 11.5254 vs 3.3930, *P* < 0.001, Fig. 4A) and a higher ratio of CD39^**+**^Foxp3^**+**^/Foxp3^**+**^ (83.34% vs 79.19%, *P* = 0.013).

### 3.6 Prognostic significance of Foxp3^+^ and CD39^+^Foxp3^+^Tregs in HCC

By using the “minimum *P* value” approach, the counts of 2.33 vs 0.67 and 2.00 vs 1.33 were the best cut-off values for Foxp3^**+**^ vs CD39^**+**^Foxp3^**+**^Treg cells in tumoral and peritumoral tissues, respectively. On univariate analysis, the tumoral Foxp3^**+**^ and CD39^**+**^Foxp3^**+**^Treg cell counts were related to both TTR and OS (Table 2, Fig. 3E-H), and so were the peritumoral Foxp3^+^Treg counts (Table 2). Multivariate Cox proportional hazard regression analyses showed that the levels of intratumoral Foxp3^**+**^ and CD39^**+**^Foxp3^**+**^Tregs had a prognostic role in TTR and OS. Furthermore, CD39^+^Foxp3^+^Tregs were a better prognosticator than Foxp3^+^Tregs for TTR (HR hazard ratio [HR] = 1.708 vs HR = 1.546).

## 4 Discussion

In this study, we report for the first time that CD39 can be detected immunohistochemically on tumor cells, endothelial cells, macrophagocytes, and Tregs in HCC. Compared with paired peritumoral tissues, tumoral tissues had significantly higher expression of CD39, more Foxp3^+^ and CD39^+^Foxp3^+^Treg cell counts and a higher ratio of CD39^+^Foxp3^+^ to Foxp3^+^Treg cells. The levels of CD39 expression were related to both TTR and OS. Furthermore, the intratumoral Foxp3^+^ and CD39^+^Foxp3^+^Treg cell counts had a prognostic role in TTR and OS. However, CD39^+^Foxp3^+^Tregs were a better prognosticator than Foxp3^+^Tregs for TTR.

CD39 was originally characterized as a cell activation marker, and was identified on B cells, subsets of activated NK-cells, and T-lymphocytes.^[12,26,27]^ In the liver, CD39 was detected immunohistochemically on endothelial cells of muscularized vessels, Kupffer cells, and subsets of liver lymphocytes such as NK, Natural killer T, and B cells.^[28]^ It was also a functional marker on Tregs, which links them to ATP breakdown and potentially to the production of immunosuppressive adenosine.^[29]^ In this study, we used immunohistochemical methods to determine the expression of CD39 and related markers in situ in HCC and found that CD39 was extensively expressed on endothelial cells, macrophagocytes, and Tregs as well as on tumor cells in HCC. In the tumor cell lines, the expression level of CD39 was closely associated with further pulmonary metastatic potential.

The mechanism of CD39 expression on different cells in the progression of HCC was not uniform. Some studies have demonstrated that CD39 was implicated in promoting tumor growth and metastases through the suppression of antitumor immune responses and enhancement of angiogenesis.^[29,30]^ Extracellular ATP directly limits tumor cell growth and these antitumor effects can be mitigated by provision of CD39 or by the intrinsic EC expression of CD39.^[24]^ Our study found that CD39 was mostly expressed on ECs, which probably played an important role in the progression of HCC; this is in agreement with our data on the levels of tumoral CD39 expression which were shown to be related to TTR and OS. Liao et al^[31]^ identified cAMP as a crucial regulator of macrophage CD39 expression and demonstrated that cAMP acts through the PKA/CREB, PKA/PI3K/ATF2, and PKA/ERK/ATF2 pathways to control a key vascular homeostatic mediator,^[31]^ which might explain in part the mechanism of CD39 expression on macrophages.

Tregs are key immunosuppressive cells in the context of cancer and high infiltration of Tregs correlates with a poor prognosis in most cancer types, including HCC.^[16,32,33]^ One key mechanism in immunomodulation by Tregs seems to be the generation of extracellular adenosine. CD4^+^CD25^+^FoxP3^+^Tregs were shown to use CD39 and CD73 to hydrolyze adenosine tri- and diphosphate (ATP/ADP) to adenosine, which in turn exerts immunosuppression on various immune cell populations.^[34]^ The development and immunosuppressive functions of CD4^+^CD25^+^FoxP3^+^Tregs are under the influence of the adenosine-A2A adenosine receptor pathway.^[35]^ Human Tregs characterized by the presence of CD39 and the low expressionof CD26/ADA were responsible for the generation of adenosine, which played a major role in Tregs-mediated immunosuppression.^[36]^ CD39 expression on Tregs has also been shown to inhibit NK cell activity and to promote hepatic metastasis in a murine melanoma cancer model,^[24]^ and is highly involved in mediating the suppressive activity of tumor-infiltrating CD8^+^ T regulatory lymphocytes.^[37]^ In our study, we found that the higher level of CD39^+^Foxp3^+^Tregs count was a better prognosticator than Foxp3^+^Tregs for TTR, which indicated that CD39^+^Foxp3^+^Tregs might be a activated regulatory T cells in HCC.

In humans, the expression of CD39 is not homogeneous within the FoxP3^+^ population. An increased frequency of CD4^+^CD39^+^Tregs has been reported in tumor-infiltrating T cells in lymphoma patients.^[38]^ Similar to CD39^+^Tregs in the peripheral blood, half of these cells are CD25^+^FoxP3^+^ active suppressor cells. Mandapathil et al^[39]^ found that up to 80% of human FoxP3^+^Treg cells were CD39^+^ in the peripheral blood of patients with head and neck cancer, higher than that in normal subjects. Furthermore, most CD4^+^CD25^+^ cells express CD39 *in situ*. We found that the CD39^+^Foxp3^+^/Foxp3^+^ ratio in tumors was 83.34%, higher than that in peritumoral tissues, and may be characterized as a cell activation marker.^[40]^ In this study, the extensive expression of CD39 in HCC indicated that the tumor escape mechanisms might include both tumor-derived and host-related factors.

The high expression of CD39 and Foxp3 was also reported in some other cancers, not specific to HCC. However, the mechanism of this finding remains to be further explored and it will be a big challenge that CD39 can serve as therapeutic target of HCC.

## 5 Conclusions

Taken together, the findings of the present study indicated that CD39 expression in HCC can predict postoperative HCC recurrence and survival time of patients, and highlighted the important prognostic value of CD39^+^ Tregs count in tumoral tissues. CD39 may be a new target for antitumor immunotherapy in HCC.

## Supplementary Material

Supplemental Digital Content
