# Supplementary material for: Overexpression of CD39 in hepatocellular carcinoma is an independent indicator of poor outcome after radical resection
Source: Medicine (Baltimore). 2016 Oct 7;95(40):e4989. doi: 10.1097/MD.0000000000004989 (PMC5059057; doi:10.1097/MD.0000000000004989)
Supplement: Supplemental Digital Content [file medi-95-e4989-s001.doc]

**Supplementary File**

**Immunohistochemical double staining**

Firstly, the slides were deparafﬁnized in xylene and hydrated through a graded alcohol series. Antigen retrieval was carried out in a pressure cooker for 9 min (using a standard household model) with 0.1M EDTA (pH 8). Secondly, sections were incubated with the primary antibody cocktail at 4°C overnight after being placed in blocking solution to inhibit endogenous peroxidase activity and nonspecific background staining. Thirdly, after the specific antibodies were bound to their respective antigens in the tissue sections, the rabbit and mouse primary antibodies were located by a specially formulated cocktail of secondary antibody polymers The polymer complex was then visualized with the LV Blue chromogen for AP activity and with the LV Red chromogen for HRP activity. The same method was used for the blank controls, except the primary antibodies were omitted.

**Immunofluorescence assay**

Cells cultured on glass slides were fixed by acetone for 15 min. After treating with 0.2% Triton X-100 for 2 min, the fixed cells were blocked with bovine serum albumin and stained with rabbit anti-human CD39 monoclonal antibody (1:200) at 4℃ overnight and DyLight^TM^ 488-Conjugated Goat Anti-Rabbit IgG at 37℃ for 30 min. A negative control (primary antibody omitted) was included on each slide. After rinsing in PBS, the slides were counterstained with 4,6-diamidino-2-phenylindole (Vector Laboratories, Inc.) and examined under a fluorescent microscope (Olympus BX 40).

| **Supplementary Table 1.Clinicopathologic features of the patients.** | |
| --- | --- |
| Age, y, median (range) | 53 (10 ~ 79) |
| Gender (male/female) | 275/49 |
| HBV infection (no/yes) | 24/300 |
| Liver cirrhosis (no/yes) | 34/290 |
| AFP, ng/ml, median (range) | 100.5 (0 ~ 60500) |
| γ-GT, U/L, median (range) | 56 (7 ~ 648) |
| ALT, U/L, median (range) | 39.5 (8 ~ 949) |
| Child-Pugh score (A/B) | 324/0 |
| Tumor size, cm, median (range) | 4(1.0 ~ 21.0) |
| Tumor number (single/multiple) | 283/41 |
| Tumor capsule (yes/no) | 174/150 |
| Tumor differentiation (I/II/III/IV) | 7/236/80/1 |
| Tumor thrombi (no/yes) | 238/86 |
| BCLC stage (A/B&C) | 222/102 |
| Prophylactic therapy (none/TACE/immunotherapy ^a^) | 324/0/0 |
| Post-recurrence therapy (none/TACE/regional^b^/resection) | 208/116/0/0 |
| Abbreviations: AFP, alpha-fetoprotein; γ-GT, gamma-glutamyltranspeptidase; ALT, alanine transaminase; BCLC, Barcelona Clinic Liver Cancer; TACE, transcatheter arterial chemoembolization.  a. immunotherapy: interferon-α or thymosin therapy.  b. regional: radio frequency ablation (RFA); percutaneous ethanol injection therapy (PEI) or microwave ablation (MA). | |
